# OsB_9_^−^: An Aromatic Osmium-Centered Monocyclic Boron Ring

**DOI:** 10.3389/fchem.2021.751482

**Published:** 2021-09-10

**Authors:** Rui Yu, Sudip Pan, Zhong-hua Cui

**Affiliations:** ^1^ Institute of Atomic and Molecular Physics, Key Laboratory of Physics and Technology for Advanced Batteries (Ministry of Education), Jilin University, Changchun, China; ^2^ Wilhelm Ostwald Institute for Physical and Theoretical Chemistry, Leipzig University, Leipzig, Germany; ^3^ Fachbereich Chemie, Philipps-Universität Marburg, Marburg, Germany; ^4^ Beijing National Laboratory for Molecular Sciences, Beijing, China

**Keywords:** molecular wheel, bonding, electron delocalization, dual aromaticity, electronic structure calculation

## Abstract

Transition-metal-centered monocyclic boron wheels are important candidates in the family of planar hypercoordinate species that show intriguing structure, stability and bonding situation. Through the detailed potential energy surface explorations of MB_9_^−^ (M = Fe, Ru, Os) clusters, we introduce herein OsB_9_^−^ to be a new member in the transition-metal-centered borometallic molecular wheel gallery. Previously, FeB_9_^−^ and RuB_9_^−^ clusters were detected by photoelectron spectroscopy and the structures were reported to have singlet *D*_9*h*_ symmetry. Our present results show that the global minimum for FeB_9_^−^ has a molecular wheel-like structure in triplet spin state with *C*_s_ symmetry, whereas its heavier homologues are singlet molecular wheels with *D*_9*h*_ symmetry. Chemical bonding analyses show that RuB_9_^−^ and OsB_9_^−^ display a similar type of electronic structure, where the dual σ + π aromaticity, originated from three delocalized σ bonds and three delocalized π bonds, accounts for highly stable borometallic molecular wheels.

## Introduction

The pure and doped boron clusters have attracted great attentions because of their novel structures, intriguing chemical bonds and promising building blocks for boron-based nanomaterials (Alexandrova et al., 2006; Jian et al., 2019). Up to date, great achievements of boron-based clusters have been attained by extensive experimental and theoretical studies (Albert and Hillebrecht, 2009). They show a zoo of structural diversity ranging from planar (Pan et al., 2008; Piazza et al., 2014; Bai et al., 2019) or quasi-planar (Popov et al., 2013) configurations, tubular nanostructures (Kiran et al., 2005; Yang et al., 2008) to all-boron borospherenes/borophenes (Wang 2016; Li et al., 2017) with the increasing B_n_ size. On the other hand, the striking electronic properties, i.e., multiple aromaticity, nuclear dynamics, hydrocarbon analogues strongly enrich our knowledge of electronic theory. These unusual structural and electronic properties can be regarded as a consequence of the electron deficiency of boron atom, which gives rise to the extraordinary ability of boron to form delocalized multi-center bonds with itself and other elements. Indeed, the introduction of heteroatoms in boron clusters has created a variety of intriguing doped boron clusters, including metal-centered monocyclic ring/tubular/cage structures, (Romanescu et al., 2011; Jian et al., 2016; Dong et al., 2018; Liang et al., 2018; Chen et al., 2019; Lu et al., 2021), half-sandwich structures, (Chen et al., 2018; Ren et al., 2019), inverse sandwich structures, (Cui et al., 2020; Jiang et al., 2021), metallo-borophenes (Li et al., 2016; Zhang et al., 2016) and metallo-borospherenes, (Chen et al., 2020; Zhang et al., 2021), strongly leading to a new direction of research on boron chemistry and pushing the limit of structural chemistry as well as the record of coordination number in 2D and 3D environments for central metal atoms. (Islas et al., 2007; Liu et al., 2007; Miao et al., 2009; Li et al., 2012; Popov et al., 2014; Pan et al., 2018; Chen et al., 2019).

Amongst, the metal-centered monocyclic wheels represent a family of fascinating planar double aromatic borometallic compounds (Luo 2008; Pu et al., 2009; Romanescu et al., 2013; Romanescu et al., 2013). Such species were firstly found in the global minimum of CoB_8_^−^ and FeB_9_^−^ predicted by computational studies (Ito et al., 2008; Pu et al., 2009). After that, a set of MB_n_^−^ monocyclic wheels (CoB_8_^−^, FeB_8_^−^, FeB_9_^−^, RuB_9_^−^, RhB_9_^−^ and IrB_9_^−^) (Ito et al., 2008; Luo 2008; Romanescu et al., 2011; Li et al., 2012; Yang et al., 2015) have been characterized by the photoelectron spectroscopy supported by the computational studies. Thereafter, TaB_10_^−^ and NbB_10_^−^, the largest member setting the new limit of maximum coordination number in planar form, were also experimentally detected (Galeev et al., 2012; Li et al., 2013). The extraordinary stability in planar structures in all these metal-centered monocyclic wheels can be rationalized by the presence of σ and π double aromaticity, making it an effective electronic design principle.

We noted that MB_n_^−^ (M = group 8 and 9 elements) clusters have been detected and characterized to be the global monocyclic wheels except for M = Os. Thus, the question remains as to whether OsB_9_^−^ is a real exception. To address this issue, the detailed potential energy surfaces (PESs) of MB_9_^−^ (M = Fe, Ru, Os) were explored herein, and structural and electronic properties of the lowest-energy structures were systematically analyzed by coupling with various chemical bonding approaches. Interestingly, we found a new global minimum for FeB_9_^−^. A molecular wheel-like structure in triplet spin state with *C*_s_ symmetry is lower in energy than the previously reported singlet molecular wheel form with *D*_9*h*_ symmetry (Romanescu et al., 2012). On the other hand, OsB_9_^−^ is a singlet global monocyclic wheel that behaves similarly to RuB_9_^−^, where σ and π double aromaticity (three delocalized σ bonds and three delocalized π bonds) gives rise to their high stability, making it a suitable target for future experimental detection. (Romanescu et al., 2011).

## Computational Methods

The CALYPSO (Wang et al., 2016) (Crystal structure AnaLYsis by Particle Swarm Optimization) code was used for the detailed structural explorations of MB_9_^−^ (M = Fe, Ru, Os) in their singlet, triplet, and quintet spin states at the PBE0/def2-SVP level. For the low-lying energy isomers, further reoptimization followed by harmonic vibrational frequency calculation were done at the PBE0/def2-TZVPP level. For comparison, another level of theory, TPSSh/def2-TZVPP was also chosen. For further energetic refinement, singlet point calculations were further done at the CCSD(T) (Pople et al., 1987)/def2-TZVPP//PBE0/def2-TZVPP level. Total energies were corrected by the zero-point corrected energies (ZPE) of PBE0/def2-TZVPP level. The natural bond orbital (NBO), (Glendening et al., 2019), nucleus-independent chemical shift (NICS), (Mitchell 2001), adaptive natural density partitioning (AdNDP), (Zubarev and Boldyrev, 2008), quantum theory of atoms in molecules (QTAIM) and electron localization (ELF) analyses (Fuster et al., 2000) were performed for these global monocyclic molecular wheels using Multiwfn code (Lu and Chen, 2012). To facilitate future experimental characterization, the simulated photoelectron spectra of RuB_9_^−^ and OsB_9_^−^ were calculated at the BP86/def2-TZVPP level based on generalized Koopmans’ theorem (Tsuneda et al., 2010). The aromaticity was understood by the gauge including magnetically induced current (GIMIC) analysis (Fliegl et al., 2011) and the anisotropy of the current induced density (ACID) (Geuenich et al., 2005). All the calculations were performed using the Gaussian 09 package. (Frisch et al., 2016).

### Structures and Energetics

**FIGURE 1:**
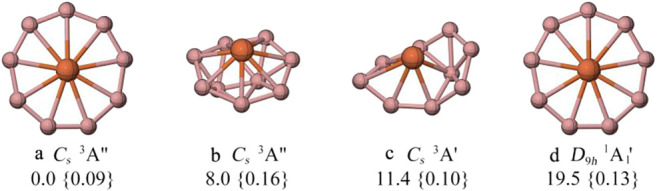
The low-lying energy isomers of FeB_9_^−^ computed at the PBE0/def2-TZVPP level and T1 diagnostic values obtained with coupled-cluster wavefunction are given in curly braces. All energies are corrected from zero-point energies (ZPE) at the PBE0/def2-TZVPP level.

The singlet PES of FeB_9_^−^ was explored in 2008, (Ito et al., 2008), where the singlet *D*_9*h*_-symmetry planar nonacoordinate Fe-centered monocyclic boron wheel (isomer **d** in Figure 1) was reported to be the lowest-energy structure that lies 14.9 kcal/mol more stable than the second alternative at the BP86/TZVPP level. In 2012, the photoelectron spectroscopy of FeB_9_^−^ was explained based on the singlet wheel isomer (Romanescu et al., 2012). However, by the detailed structural searches of singlet, triplet, and quintet states, we found that the triplet molecular wheel with *C*_s_ symmetry (**a**) is 19.5 kcal/mol lower in energy than **d** at the PBE0/def2-TZVPP level. Meanwhile, large T1 diagnostic values obtained with the coupled-cluster wave function indicate that FeB_9_^−^ system is a multireference problem. Note that the broken-symmetry spin-unrestricted approach was used for the monocyclic boron wheel, which is still 2.1 kcal/mol lower in energy relative to the closed-shell one. Thus, the coexistence of triplet global state of the molecular wheel FeB_9_^−^ could be the reason of the observed broad features in photoelectron spectrum, as assumed by the authors. (Romanescu et al., 2012).

**FIGURE 2:**
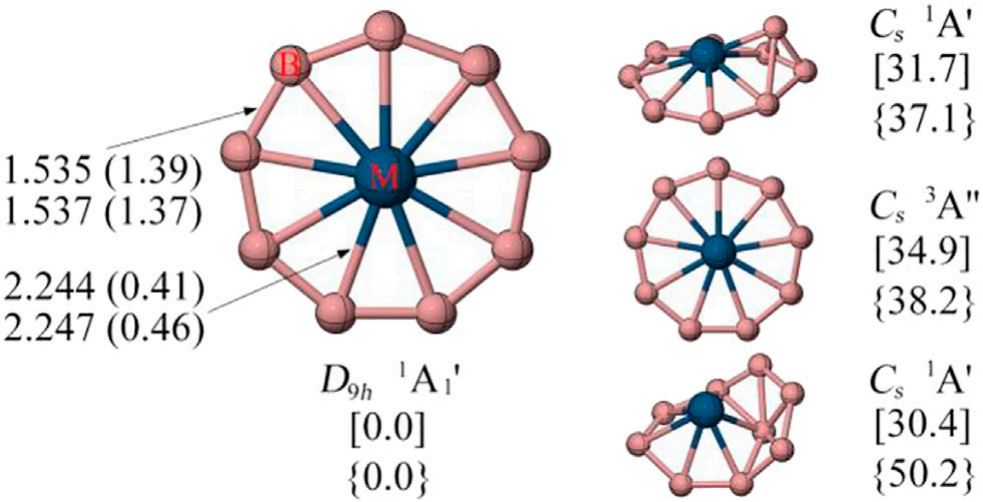
The minimum energy geometries and the corresponding bond distances in Å and WBI values in parentheses of RuB_9_^−^ **(top)** and OsB_9_^−^ **(bottom)** are given at the PBE0/def2-TZVPP level. Relative energies in kcal/mol of the low-lying energy isomers of [RuB_9_^−^] and {OsB_9_^−^} are given at the CCSD(T)/def2-TZVPP//PBE0/def2-TZVPP level with ZPE correction of PBE0. The T1 diagnostic are less than 0.05.

Figure 2 displays the low-lying energy isomers of RuB_9_^−^ and OsB_9_^−^. The monocyclic boron wheel with *D*_9*h*_ symmetry and ^1^A_1_^’^ electronic state is predicted to be a real global minimum having the lowest vibrational frequencies of 62.2 and 17.2 cm^−1^ for RuB_9_^−^ and OsB_9_^−^, respectively. At the CCSD(T)/def2-TZVPP level, the monocyclic boron wheel is a global minimum that lies 30.4 and 37.1 kcal/mol more stable than the second alternative for RuB_9_^−^ and OsB_9_^−^, respectively. The triplet monocyclic boron wheels are also located, but unlike FeB_9_^−^, they are significantly high-energy isomers. Note that the results at the TPSSh/def2-TZVPP level are very similar to the PBE0/def2-TZVPP level, except for the relative energy between isomer **a** and **d** of FeB_9_^−^ (see Supplementary Figure S1). This is presumably because of the multireference character in these systems. The T1 diagnostic factors of RuB_9_^−^ and OsB_9_^−^ are within 0.05, suggesting that the single-reference method can be safely used for these two clusters. Given the fact that RuB_9_^−^ was detected earlier by photoelectron spectroscopy, we believe that the monocyclic boron wheel OsB_9_^−^ cluster is also a suitable target for the gas-phase experimental study.

To understand the high stability of the MB_9_^−^ monocyclic wheels, their detailed structural parameters are given in Figure 2. We found the MB_9_^−^ (M = Ru, Os) clusters possess similar structural properties. In the case of OsB_9_^−^, like all other metal-centered monocyclic boron wheels, the B-B bonds show strong multiple bonding characteristic as indicated by the short bond distance of 1.54 Å and Wiberg bond indices (WBIs) value of 1.37, which is clearly shorter than the single B-B bond (1.70 Å) using the self-consistent covalent radius of Pyykkö (Pyykko and Atsumi, 2009). The strong peripheral B-B bonds is because each boron atom fully participate in the two-center two electron (2c-2e) B-B σ bonds and two sets of the delocalized σ and π bonds (see discussed below). The M-B bonds of OsB_9_^−^ have the bond distance of 2.247 Å (WBI = 0.46), which is slightly longer than the M-B single bond using the self-consistent covalent radius of Pyykkö, a common characteristic for the multicentered bonds. (Pyykko and Atsumi, 2009).

### Electronic Delocalization

**FIGURE 3:**
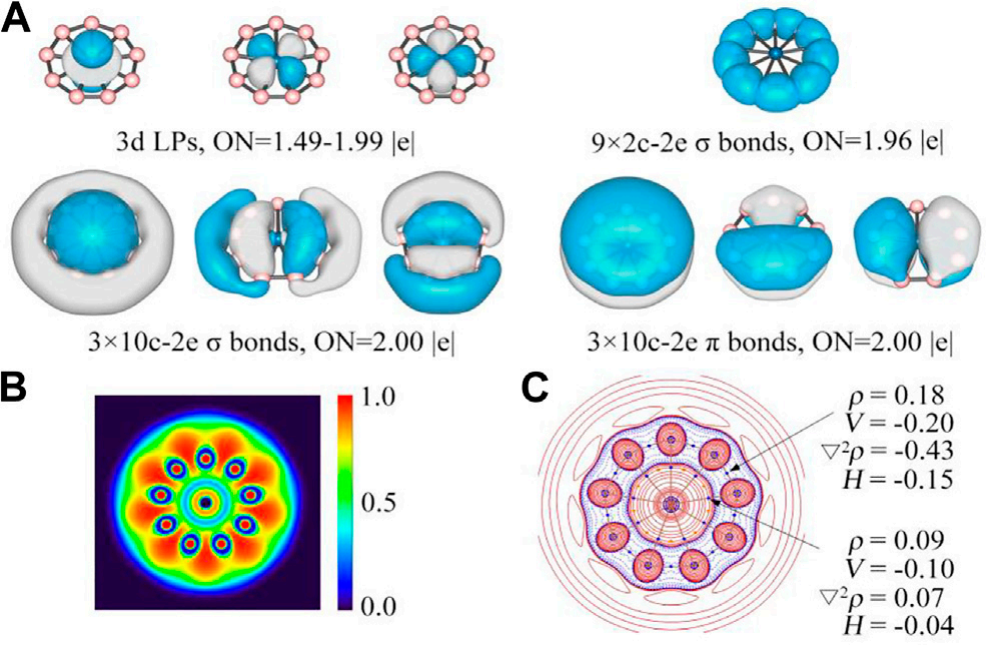
**(A)** AdNDP results, and **(B)** color-filled map of ELF and **(C)** contour plot of Laplacian of electron density of OsB_9_^−^. In c, the contour line map of Laplacian of electron density, red solid lines and blue dotted lines represent positive and negative regions, respectively. Blue and orange points correspond to position of bond critical points (BCPs) and ring critical point (RCP), respectively. Values of some real space functions at the BCP are given, including ρ (electron density), *V* (potential energy density), ∇^2^ *ρ*, *H* (energy density).

The adaptive natural density partitioning (AdNDP) (Zubarev and Boldyrev, 2008) analyses were carried out for OsB_9_^−^ to further understand its chemical bonding and electronic structure. As shown in Figure 3A, the first row displays three one center-two electrons (1c-2e) lone pair electrons associated with d orbitals of Os center, where the occupation number (ON) for the d_z_^2^ LP is 1.99 |e| and the same for others two are 1.49 |e|. Somewhat lower ON for these LPs are because of partial delocalization to boron rings. An alternative 10c-2e description gives ideal 2.00 |e| ON, but we continue it as 1c-2e LPs for similarity since in the previously reported AdNDP results for RuB_9_^−^ the authors describe them as LPs (Romanescu et al., 2011). Nevertheless, even consideration of them as 10c-2e delocalized σ-bonds would not change the nature of aromaticity drawn based on the number of delocalized electrons. Nine 2c-2e bonds with ONs of 1.96 |e| account for the peripheral B-B bonds. The second row presents three delocalized 10c-2e σ bonds (left) and three delocalized 10c-2e π bonds (right), and they vividly satisfy the σ + π double aromaticity. The electron localization function (ELF) (Fuster et al., 2000) as shown in Figure 3B further confirms AdNDP results. The plot of ELF shows that the strong electron density is localized in the peripheral boron ring, but relatively lower electron density between M center and boron ring because of the delocalized σ and π clouds.

We performed quantum theory of atom in molecules (QTAIM) analysis to shed additional light into the nature of Os-B interaction. The contour plot of Laplacian of the electron density (∇^2^ *ρ*(**r**)) at the molecular plane is given in Figure 3C. There are nine bond paths and bond critical points (indicated by the small blue spheres) between Os and boron centers. The plot also shows that there are electron density accumulated regions (indicated by blue dotted lines) in between B and Os centers but BCPs just lie outside of the blue dotted regions because of polar nature of the bond giving positive ∇^2^ *ρ*(**r**_**c**_) value at BCP. This is a very usual feature for the bonds involving heavier elements where the criterion of negative ∇^2^ *ρ*(**r**_**c**_) value at BCP for covalent bond does not satisfy. For these cases, the total energy density *H*(**r**_**c**_) is more suitable descriptor for such cases which is negative for covalent bonds (Cremer and Kraka, 1984).^55^ The corresponding value of *H*(**r**_**c**_) at the BCP of Os-B bonds is −0.04 au, showing their covalent nature. On the other hand, for B-B bonds as expected both ∇^2^ *ρ*(**r**_**c**_) and *H*(**r**_**c**_) are negative. Similar electron topology is noted in case of RuB_9_^−^ as well (see Supplementary Figure S2 in supporting information).

### Aromaticity

**FIGURE 4:**
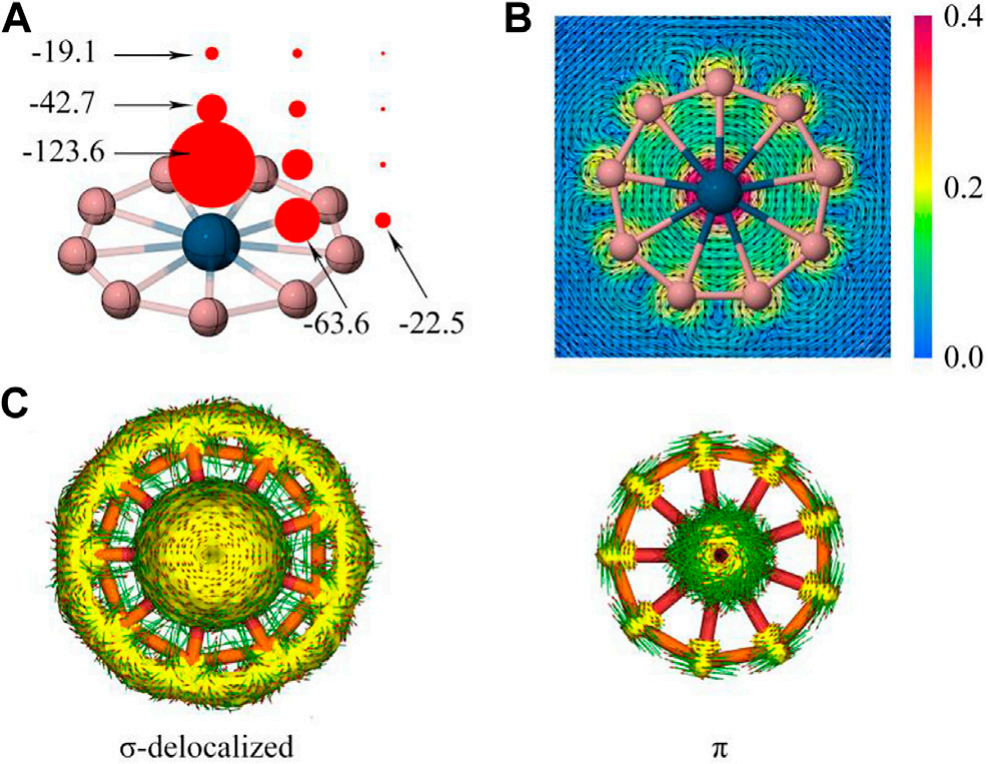
**(A)** NICS_zz_, and **(B)** GIMIC map and **(C)** induced ring current of the delocalized σ and π electrons based on ACID values of OsB_9_^−^. In b), the arrows indicate direction of induced current, the color correspond to magnitude of induced current.

The dual σ + π aromaticity was further confirmed in the following discussion. The nucleus-independent chemical shift (NICS) (Mitchell 2001) is a key method to quantify aromaticity, where NICS_zz_ values (the out-of-plane (“zz”) shielding tensor component of NICS). As shown in Figure 4A, the grids of NICS_zz_ points are created at the center of wheels, the center of B-M-B ring and out of the ring associated with 1.0 Å vertical spacings from the wheel plane. The considerable negative NICS_zz_ values vividly show aromatic boron wheels, especially the big NICS(1)_zz_ of the wheel centers (−123.6 ppm) is consistent with the reported transition-metal-centered borometallic molecular wheel family. Figure 4B displays a gauge including magnetically induced current (GIMIC) map, (Fliegl et al., 2011), where the induced ring current is generated by employing an external magnetic field perpendicular to the molecular plane. The diatropic (clockwise) current comply with the left-handed rule. It is worthy of note that the inner and outside of the peripheral ring both show a diatropic and unidirectional current. This current behavior is similar to the C_18_ clusters with double aromaticity (σ + π) but sharply different from the benzene (π aromaticity only), where the ring current show a diatropic inside but paratropic outside of benzene ring. The induced current density (**J** ^ind^) is integrated into a specific area, which starts at the center of the ring and intersects the B-B bond ending about 4 Å away for the current system. The ring-current strength of RuB_9_^−^ (25.4 nA/T) and OsB_9_^−^ (26.4 nA/T) is similar to C_18_ (Lu et al., 2020) (25.3 and 21.2 nA/T), and stronger than the benzene (11.5 nA/T) at the wB97XD/def2-TZVP level, which could be another indicator of dual σ + π aromaticity. The anisotropy of the current induced density (ACID) is able to describe the σ and π contribution for aromaticity as given in Figure 4C. Overall, the σ and π dual aromaticity is strongly confirmed by these analyses in Figure 4 and Supplementary Figure S3 for OsB_9_^−^ and RuB_9_^−^, respectively.

### Simulated Photoelectron Spectra

**FIGURE 5:**
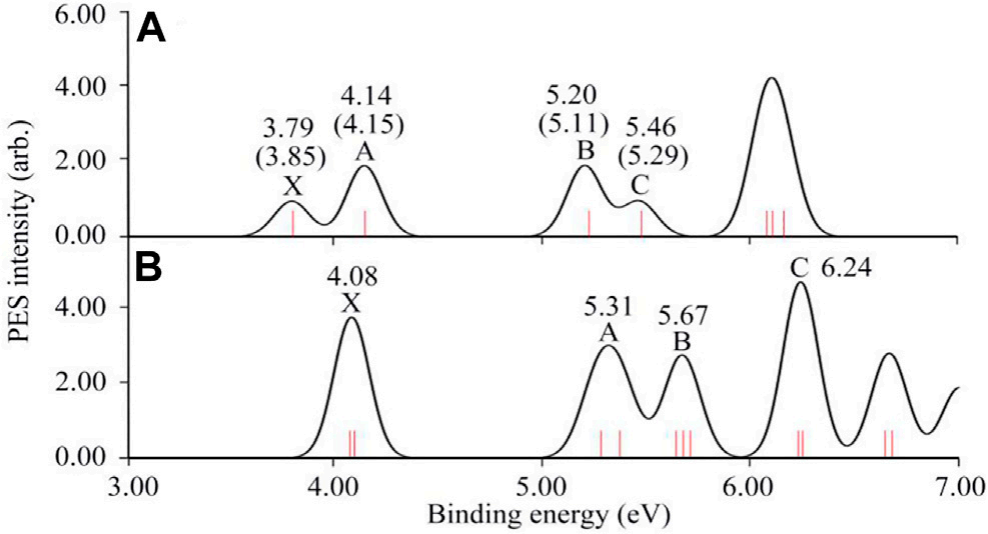
The simulated photoelectron spectrums of **(A)** RuB_9_^−^ and **(B)** OsB_9_^−^ were calculated at the BP86/def2-TZVPP level based on generalized Koopmans’ theorem. The experimental results of RuB_9_^−^ are given in parenthesis.

The simulated photoelectron spectra of RuB_9_^−^ and OsB_9_^−^ are given in Figure 5 based on the generalized Koopmans’ theorem (Tsuneda et al., 2010). The simulated spectrum for RuB_9_^−^ is in good agreement with the experimental data as shown in Figure 5A. Thus, to facilitate the experimental confirmation, the simulated photoelectron spectrum of OsB_9_^−^ cluster is illustrated in Figure 5B, where the well-resolved detachment transitions at the lower-binding-energy side, are labeled as X (4.08), A (5.31), B (5.67), C (6.24) in eV.

## Conclusion

The OsB_9_^−^ cluster was found to be a new member of transition-metal-centered borometallic molecular wheel family. The detailed electronic structure analyses including the AdNDP, ELF, NICS, and ACID approaches all suggested that the dual σ + π aromaticity (three delocalized σ bonds and three delocalized π bonds) occurs in RuB_9_^−^ and OsB_9_^−^, and it is a key factor to design highly stable borometallic molecular wheels. Additionally, we found a different picture relative to the previous work for FeB_9_^−^. The present results show that the global minimum for FeB_9_^−^ has a molecular wheel-like structure in triplet spin state with *C*_s_ symmetry, whereas previously reported singlet molecular wheels with *D*_9*h*_ symmetry is higher energy isomer.

## Data Availability

The original contributions presented in the study are included in the article/Supplementary Material, further inquiries can be directed to the corresponding authors.
