# Supplementary material for: OsB9 −: An Aromatic Osmium-Centered Monocyclic Boron Ring
Source: Front Chem. 2021 Sep 10;9:751482. doi: 10.3389/fchem.2021.751482 (PMC8460756; doi:10.3389/fchem.2021.751482)
Supplement: Supplementary file 1 [file DataSheet1.docx]

**Supporting Information**

## **OsB_9_^−^: An Aromatic Osmium-Centered Monocyclic Boron Ring**

Rui Yu,^a^ Sudip Pan,*^,b,c^ Zhong-hua Cui,*^a,d^

*^a^Institute of Atomic and Molecular Physics, Key Laboratory of Physics and Technology for Advanced Batteries (Ministry of Education), Jilin University, Changchun, China*

*E-mail: [zcui@jlu.edu.cn](mailto:zcui@jlu.edu.cn)*

*^b^Wilhelm Ostwald Institute for Physical and Theoretical Chemistry, Leipzig University, Linnéstraße 2, D-04103, Leipzig*

*^c^Fachbereich Chemie, Philipps-Universität Marburg, Hans-Meerwein-Strasse 4, 35032 Marburg, Germany*

*E-mail:* [*pans@chemie.uni-marburg.de*](mailto:pans@chemie.uni-marburg.de)

*^d^Beijing National Laboratory for Molecular Sciences*

**
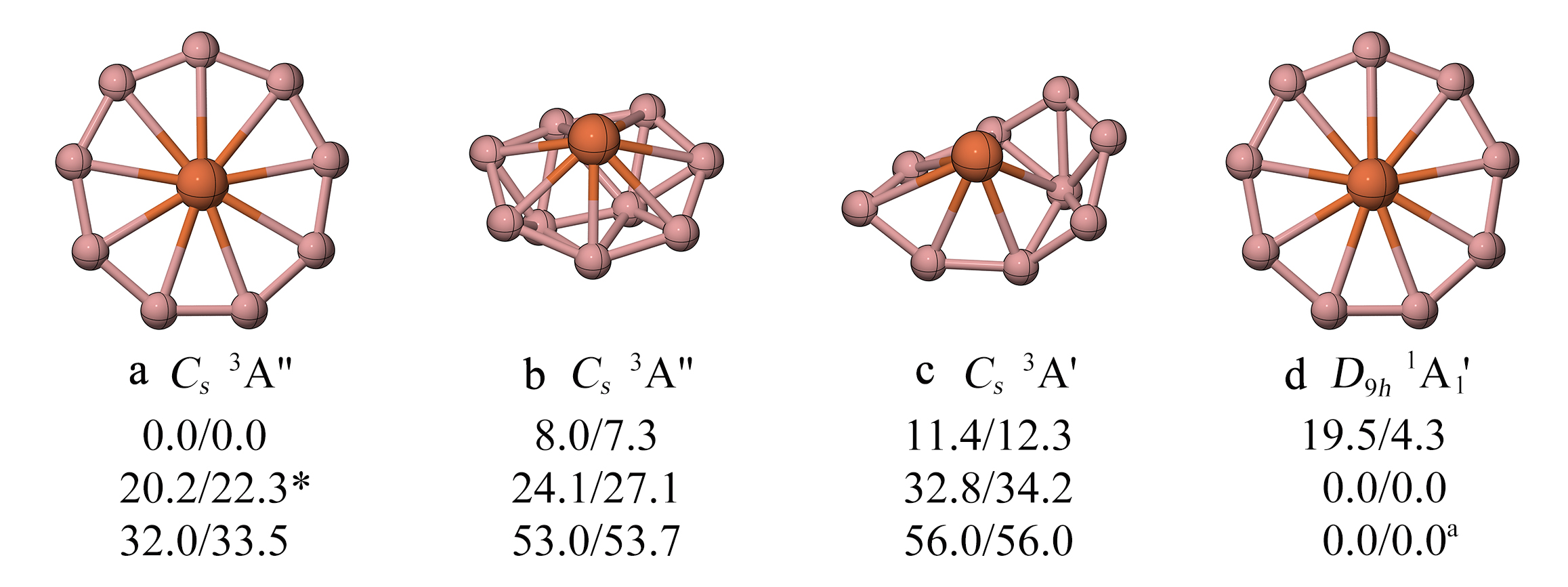
**

**Figure S1.** Optimized structures and relative energies (kcal/mol) of FeB_9_^−^, RuB_9_^−^ and OsB_9_^−^ (from top to bottom) using PBE0/TPSSh methods with def2-TZVPP basis set. The relative energies are corrected by zero-point energy. *indicates a small imaginary frequency. ^a^*C_s_ symmetry*.

**
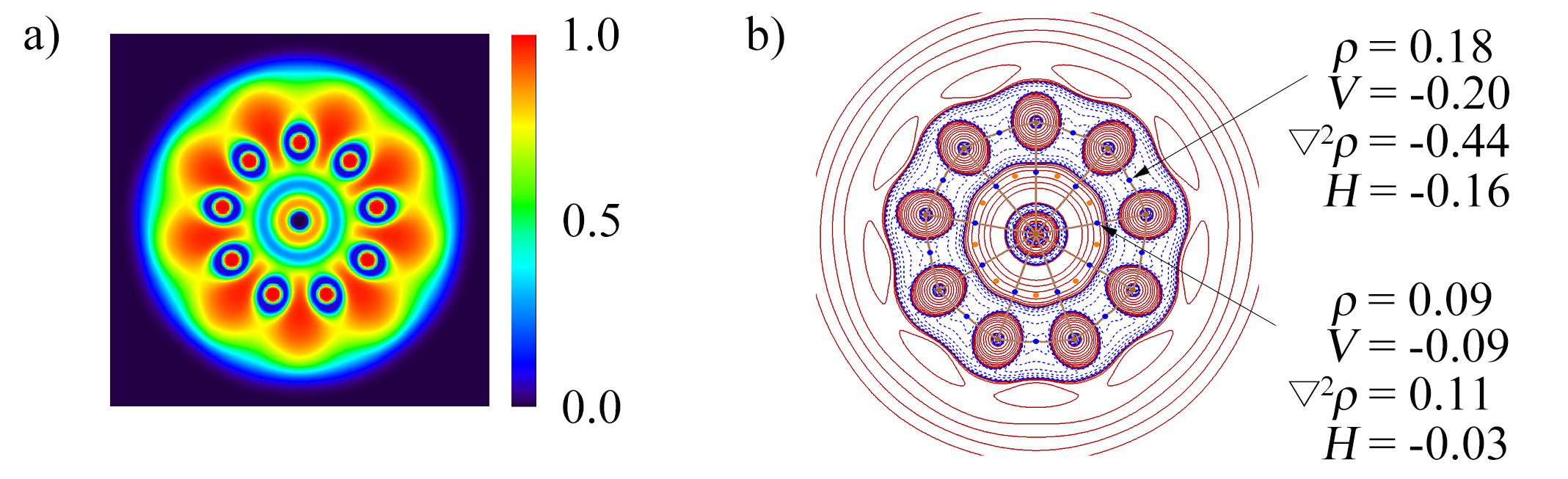
**

**Figure S2.** a) ELF analysis and b) plot of Laplacian of electron density of RuB_9_^−^. In b, the contour line map of Laplacian of electron density (∇^2^*ρ*), red solid lines and blue dashed lines represent positive and negative regions, respectively. Blue and orange points correspond to position of bond critical points (BCPs) and ring critical point (RCP), respectively. Values of some real space functions at the BCP are given, including *ρ* (electron density), *V* (potential energy density), ∇^2^*ρ*, *H* (energy density).

**
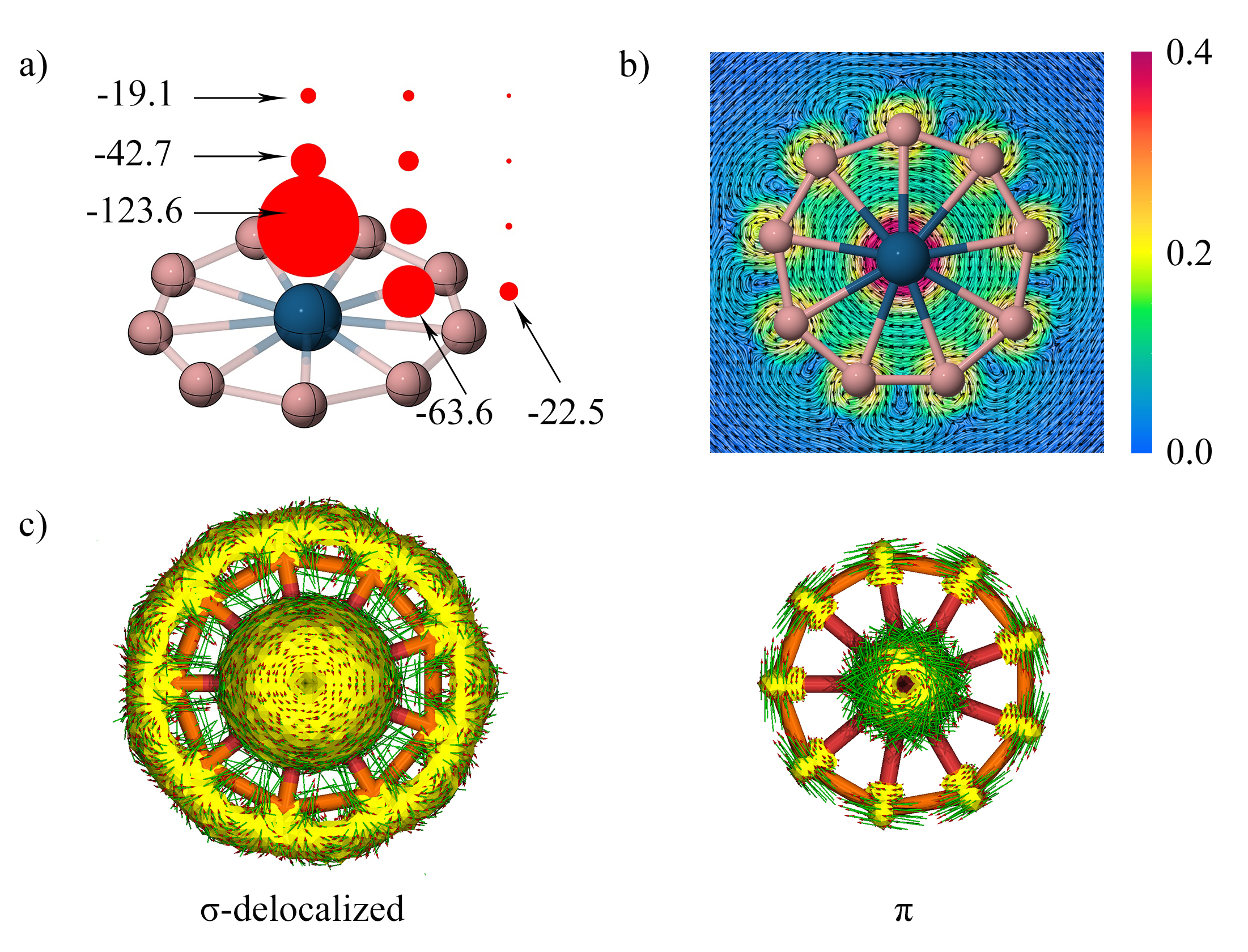
**

**Figure S3**. a) NICS_zz_, and b) GIMIC map and c) induced ring current of the delocalized σ and π electrons based on ACID values of RuB_9_^−^. In b), the arrows indicate direction of induced current, the color correspond to magnitude of induced current.

**I**. Coordinates obtained at the PBE0/def2-TZVPP level.

FeB_9_^−^ *C_s_*

5 0.000000000 -0.021180000 -2.269239000

5 0.000000000 1.410771000 -1.712844000

5 0.000000000 -1.910284000 1.107661000

5 0.000000000 1.939649000 1.151172000

5 0.000000000 2.180957000 -0.370503000

5 0.000000000 -0.741112000 2.116228000

5 0.000000000 -2.191238000 -0.413069000

5 0.000000000 0.785580000 2.145671000

5 0.000000000 -1.453134000 -1.755058000

26 0.000000000 -0.000011000 -0.000027000

OsB_9_^−^ *D*_9_*_h_*

5 0.000000000 2.246590000 0.000000000

5 1.945604000 -1.123295000 0.000000000

5 -1.945604000 -1.123295000 0.000000000

5 0.768379000 -2.111104000 0.000000000

5 1.444080000 1.720988000 0.000000000

5 -0.768379000 -2.111104000 0.000000000

5 2.212460000 0.390116000 0.000000000

5 -2.212460000 0.390116000 0.000000000

5 -1.444080000 1.720988000 0.000000000

76 0.000000000 0.000000000 0.000000000

RuB_9_^−^ *D*_9_*_h_*

5 1.943344000 1.121990000 0.000000000

5 -1.442403000 -1.718989000 0.000000000

5 -1.943344000 1.121990000 0.000000000

5 0.767487000 2.108652000 0.000000000

5 2.209890000 -0.389663000 0.000000000

5 0.000000000 -2.243981000 0.000000000

5 1.442403000 -1.718989000 0.000000000

5 -2.209890000 -0.389663000 0.000000000

5 -0.767487000 2.108652000 0.000000000

44 0.000000000 0.000000000 0.000000000
